# The paradigm of tax-reward and tax-punishment strategies in the advancement of public resource management dynamics

**DOI:** 10.1098/rspb.2024.0182

**Published:** 2024-06-12

**Authors:** Lichen Wang, Yuyuan Liu, Ruqiang Guo, Liang Zhang, Linjie Liu, Shijia Hua

**Affiliations:** ^1^ College of Science, Northwest A&F University, Yangling 712100, People’s Republic of China; ^2^ College of Economics and Management, Northwest A&F University, Yangling 712100, People’s Republic of China

**Keywords:** evolutionary game theory, common resource, tax-reward, tax-punishment

## Abstract

In contemporary society, the effective utilization of public resources remains a subject of significant concern. A common issue arises from defectors seeking to obtain an excessive share of these resources for personal gain, potentially leading to resource depletion. To mitigate this tragedy and ensure sustainable development of resources, implementing mechanisms to either reward those who adhere to distribution rules or penalize those who do not, appears advantageous. We introduce two models: a tax-reward model and a tax-punishment model, to address this issue. Our analysis reveals that in the tax-reward model, the evolutionary trajectory of the system is influenced not only by the tax revenue collected but also by the natural growth rate of the resources. Conversely, the tax-punishment model exhibits distinct characteristics when compared with the tax-reward model, notably the potential for bistability. In such scenarios, the selection of initial conditions is critical, as it can determine the system’s path. Furthermore, our study identifies instances where the system lacks stable points, exemplified by a limit cycle phenomenon, underscoring the complexity and dynamism inherent in managing public resources using these models.

## 1. Introduction

The rampant overexploitation of public resource pools has emerged as a pervasive issue in today’s world, cutting across geographical and cultural boundaries [1–4]. As humanity’s footprint on ecosystems escalates, the reciprocal influence of ecological variables on human societies is concurrently intensifying. This intricate interplay between societal and ecological systems is not an isolated incident but a globally prevalent phenomenon [5–8]. A case in point is the widespread overgrazing of public rangelands. Across numerous regions, rangeland resources are subjected to relentless exploitation, culminating in their alarming depletion [9,10]. This predicament frequently arises in the absence of effective management and vigilant supervision, as herders, driven by their necessities, overgraze the land, thereby impeding the resources’ ability to rejuvenate and propelling them towards eventual exhaustion. This scenario is not confined to rangelands alone but is mirrored in a multitude of contexts, such as the overfishing of marine resources, the excessive consumption of water resources, and the exacerbation of global climate change [11–15]. These instances underscore the urgent need for comprehensive strategies to manage public resources sustainably.

To address the challenge of resource overexploitation, managers often establish allocation rules. Despite these efforts, individual desires for greater benefits frequently lead to the violation of these rules, exacerbating resource depletion. This scenario underscores the significance of cooperative behaviour, wherein participants comply with allocation rules out of a collective commitment to resource sustainability. This raises a pivotal question: how can we motivate individuals to persist in such cooperative behaviour for the enduring sustainability of resources? This inquiry has generated substantial interest and debate in the field [16–19].

The intricacies of public resource management often call for the adoption of reward and punishment strategies, a stance substantiated by a wealth of studies [20–30]. In particular, the work of Chen & Szolnoki highlights the effectiveness of punitive mechanisms [31]. Their inspection–punishment model, which targets and penalizes defectors, demonstrates how different natural growth rates impact the evolution of public resource pools. Yet, this model applies a constant severity of punishment, while in reality, levels of punishment often adjust in response to varying violation rates. The dynamic incentive allocation mechanism can address these shortcomings. Under this scheme, all individuals pay taxes. In the reward scenario, the total tax collected is evenly distributed among all cooperators as a reward [32,33]. In the punishment scenario, the total tax is evenly allocated to the defectors as a penalty [34,35]. However, the use of this dynamic incentive allocation mechanism is seldom mentioned or employed in research on public pool management. Therefore, its effectiveness in promoting the emergence of cooperation and ensuring sustainable resource use remains to be seen.

This study pioneers the application of a tax incentive strategy to the management of public resource pools, using replicator equations to portray the evolutionary dynamics of cooperation [36–40]. Concurrently, a modified logistic model is employed to depict the evolutionary dynamics of public resource pools [40,41]. We construct tax-reward and tax-punishment models to offer a more comprehensive understanding and prediction of the temporal evolution of public resource pools. We take into account the finite and renewable nature of the resource pool, along with the reciprocal influence of individual behaviour on resources. For the tax-reward strategy, we demonstrate the temporal evolution of the proportion of cooperators and track the changes in the common resource pool, capturing the dynamics associated with varying tax revenues and natural growth rates. Our findings reveal that the system may ultimately gravitate towards one of three states: full cooperation, full defection, or a coexistence of cooperation and defection. For the tax-punishment strategy, we similarly demonstrate the temporal evolution of the proportion of cooperators and track the changes in the common resource pool, capturing the dynamics associated with varying tax revenues and natural growth rates. Contrary to the tax-reward strategy, we observe that the system may exhibit bistability, suggesting that the stability of the system is contingent upon initial conditions. Furthermore, we identify instances where stable fixed points do not exist, such as the limit cycle discussed in this paper, representing a case of unstable fixed points. Lastly, we corroborate the theoretical accuracy and the efficacy of our model through numerical examples and Monte Carlo simulations.

## 2. Model and methods

Let us envision a group of *N* individuals, all using a shared, finite and renewable resource pool, with a natural growth rate denoted as *r*. The current resource level of the common pool is represented by *y*. Typically, the logistic model is used to describe the dynamic changes [31]. The equation for the current resource total *y* with respect to time *t* is as follows:

1.1 $$\dot{y}=ry\left(1-\frac{y}{R_{m}}\right),$$

where $\dot{y}=dy/dt$ and *R*_m_ represent the maximum capacity of the resource environment. To ensure the sustainable development of the common resource, a distribution rule is proposed in [31]. Each individual obtains a resource quantity of *b*_*l*_ = *b*_m_*y*/*R*_m_ from the current resource pool, where *b*_m_ specifies the maximum resource amount an individual is allowed to use when the resource reaches its maximum capacity.

To streamline our model, we posit that individuals adopt one of two possible strategies. The first strategy, undertaken by law-abiders or cooperators, involves adhering to established allocation rules. Conversely, the second strategy is employed by law-breakers or defectors, who disregard these rules in favour of personal gain, securing a portion, *b*_v_, that surpasses the designated quota, *b*_l_. We define *b*_v_ as *b*_l_(1 + *α*), with *α* signifying the ‘defection rate’, akin to the dilemma strength often mentioned in prior research on 2 × 2 and *N*-player games [42–45]. In this scenario, we presume that an individual’s payoff is equivalent to the share of resources they extract from the collective resource pool.

In analysing a well mixed population, we employ replicator equations to model the temporal evolution of competing strategies. The fraction of cooperators within the population is denoted by *x*; consequently, we can assert the governing equation as follows:

1.2 $$\dot{x}=x(1-x)(P_{C}-P_{D}),$$

wherein $\dot{x}$ signifies the rate of change of *x* concerning time *t*, and symbols *P*_C_ and *P*_D_ pertain to the average payoffs for cooperators and defectors, correspondingly.

As the concept of coevolution accounts for the reciprocal influence of individual behaviour on the prevailing resource conditions, the equation governing the abundance of shared resources can be extended to

1.3 $$\dot{y}=ry\left(1-\frac{y}{R_{m}}\right)-N(b_{l}x+(1-x)b_{v}).$$

To avoid cooperators turning into defectors owing to unfair distribution of benefits, leading to the depletion of common resources, we propose the following two incentive strategies to promote the sustainable development of common resources.

### (a) Tax-reward

In the context of real-world resource management, rewarding cooperators is generally considered an effective strategy. Therefore, we propose a tax-reward strategy. In this strategy, we assume a population consisting of *N* individuals, with *N*_C_ being the number of cooperators. Each individual is required to pay a tax of *δ*. The total revenue generated from these taxes is then used to provide rewards to the cooperators. The average payoff for cooperators, denoted as *P*_C_, and the average payoff for defectors, denoted as *P*_D_, can be expressed as follows:

1.4 $$\begin{array}{cc} \; & P_{C}=\sum_{k=0}^{N-1}\left(\begin{array}{c} N-1\\ k \end{array}\right)x^{k}{\left(1-x\right)}^{N-1-k}\left(b_{l}-\delta +\frac{N\delta }{k+1}\right)\;\\ \mathrm{and}\;\; & P_{D}=\sum_{k=0}^{N-1}\left(\begin{array}{c} N-1\\ k \end{array}\right)x^{k}{\left(1-x\right)}^{N-1-k}(b_{l}(1+\alpha )-\delta ).\; \end{array}\}$$

According to the property of binomial coefficients $\left(\begin{array}{c} N-1\\ k \end{array}\right)\frac{N}{k+1}=\left(\begin{array}{c} N\\ k+1 \end{array}\right)$, we can obtain

1.5 $$\begin{array}{rl} P_{C}-P_{D} & =\sum_{k=0}^{N-1}\left(\begin{array}{c} N-1\\ k \end{array}\right)x^{k}{\left(1-x\right)}^{N-1-k}\left(-b_{l}\alpha +\frac{N\delta }{k+1}\right)\\ & =-\frac{b_{m}y}{R_{m}}\alpha +\delta \sum_{k=0}^{N-1}\left(\begin{array}{c} N\\ k+1 \end{array}\right)x^{k}{\left(1-x\right)}^{N-1-k}\\ & =-\frac{b_{m}y}{R_{m}}\alpha +\delta \frac{1-{\left(1-x\right)}^{N}}{x}. \end{array}$$

Based on recent dynamic feedback modelling approaches [31,46], we derived our tax-reward model as follows:

1.6 $$\left\{ \begin{array}{c} \dot{x}=x(1-x)\left(\delta \frac{1-{\left(1-x\right)}^{N}}{x}-\frac{y}{R_{m}}b_{m}\alpha \right),\\ \dot{y}=ry\left(1-\frac{y}{R_{m}}\right)-N\frac{y}{R_{m}}b_{m}(1+(1-x)\alpha ). \end{array}\right.$$

### (b) Tax-punishment

In real-life situations, when the proportion of cooperators is relatively high, the cost of rewarding them can often be substantial. In such cases, it becomes necessary to consider the punishment of defectors. We propose a tax-punishment strategy. Each individual is required to pay a tax of *δ*. The total revenue generated from these taxes is then used to administer punishment to the defectors. Similarly, the average payoff for cooperators, denoted as *P*_C_, and the average payoff for defectors, denoted as *P*_D_, can be expressed as follows:

$$P_{C}=\sum_{k=0}^{N-1}\left(\begin{array}{c} N-1\\ k \end{array}\right)x^{k}{\left(1-x\right)}^{N-1-k}(b_{l}-\delta )$$

and

$$P_{D}=\sum_{k=0}^{N-1}\left(\begin{array}{c} N-1\\ k \end{array}\right)x^{k}{\left(1-x\right)}^{N-1-k}\left(b_{l}(1+\alpha )-\delta -\frac{N\delta }{N-k}\right).$$

According to the property of binomial coefficients $\left(\begin{array}{c} N-1\\ k \end{array}\right)\frac{N}{N-k}=\left(\begin{array}{c} N\\ k \end{array}\right)$, we can obtain

$$\begin{array}{rl} P_{C}-P_{D} & =\sum_{k=0}^{N-1}\left(\begin{array}{c} N-1\\ k \end{array}\right)x^{k}{\left(1-x\right)}^{N-1-k}\left(-b_{l}\alpha +\frac{N\delta }{N-k}\right)\\ & =-\frac{b_{m}y}{R_{m}}\alpha +\delta \sum_{k=0}^{N-1}\left(\begin{array}{c} N\\ k \end{array}\right)x^{k}{\left(1-x\right)}^{N-1-k}\\ & =-\frac{b_{m}y}{R_{m}}\alpha +\delta \frac{1-x^{N}}{1-x}. \end{array}$$

Based on the above, we obtained our tax-punishment model as follows:

1.7 $$\left\{ \begin{array}{rl} & \dot{x}=x(1-x)\left(\delta \frac{1-x^{N}}{1-x}-\frac{y}{R_{m}}b_{m}\alpha \right),\\ & \dot{y}=ry\left(1-\frac{y}{R_{m}}\right)-N\frac{y}{R_{m}}b_{m}(1+(1-x)\alpha ). \end{array}\right.$$

To help readers easily understand all the parameters and variables introduced in our work, we present them in table 1.

**Table 1.** Symbols and meanings used in this article.

symbol	meaning
*N*	number of individuals in the game group
*N*_C_	number of cooperators
*N*_D_	number of defectors
*y*	current environmental resource level
*x*	current proportion of cooperators
*α*	defection rate of defectors
*δ*	tax paid by each participant
*R*_m_	maximum resource carrying capacity of the environment
*b*_m_	maximum amount of resources allowed for an individual when resources reach maximum capacity
*b*_l_	amount of resources obtained by cooperators
*b*_v_	amount of resources obtained by defectors
*P*_C_	average payoff of cooperators
*P*_D_	average payoff of defectors

## 3. Results

### (a) Tax-reward

For the tax-reward model, using equation (1.6), there are four boundary fixed points in the system: (*x*, *y*) = (0, 0), (1, 0), (1, *R*_m_ − (*Nb*_m_/*r*)) and (0, *R*_m_ − (*Nb*_m_/*r*)(1 + *α*)). Additionally, when the conditions $\delta -\alpha b_{m}+(\alpha Nb_{m}^{2}/rR_{m})<0$ and $N\delta -\alpha b_{m}+(\alpha Nb_{m}^{2}/rR_{m})+(\alpha^{2}Nb_{m}^{2}/rR_{m})>0$ are satisfied, there exists one and only one interior fixed point (x*, y*), where 0 < x* < 1 and 0 < y* < *R*_m_. Otherwise, the system does not have an interior fixed point. We use the sign of the eigenvalues of the Jacobian matrix to determine the stability of the fixed points (detailed proof can be found in the electronic supplementary material). Now we present the evolution of cooperation proportion and resource abundance over time for different initial conditions, along with their corresponding phase diagrams, where the solid black dots represent stable fixed points, while the hollow black dots represent unstable fixed points. Here, we define *E*_C_ = *b*_m_*N*/*R_m_* and *E*_D_ = *b*_m_*N*(1 + *α*)/*R*_m_. We will discuss the following three cases based on different natural growth rates *r*.

#### (i) Slowly growing resource pool

**Figure 1.**
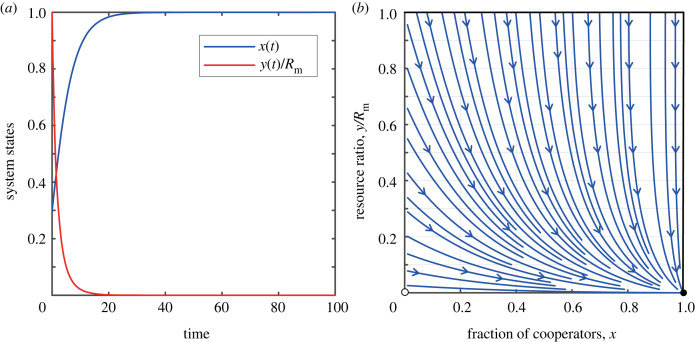
The coevolutionary dynamics of feedback-evolving game when the growth rate of the resource pool is slow. (*a*) The evolution of the system’s state over time. (*b*) The phase diagram of *x* − *y*/*R*_m_. Parameters are: *r* = 0.25, *δ* = 0.2, *N* = 1000, *R*_m_ = 1000, *α* = 0.5 and *b*_m_ = 0.5.

In the case of slow resource growth, when 0 < *r* < *E*_C_ < *E*_D_. Since *x* ∈ [0, 1] and *y* ∈ [0, *R*_m_], the system has exactly two boundary fixed points, (0, 0) and (1, 0). It is evident that, by analysing the sign of the eigenvalues, (0, 0) is unstable, while (1, 0) is stable. Figure 1 illustrates the dynamic interplay between the cooperation rate and the abundance of the shared resource pool over time, under the conditions where 0 < *r* < *E*_C_ < *E*_D_. This depiction is further complemented by an accompanying phase diagram, which effectively encapsulates the interaction of these variables within the specified parameter space. From the figure, an important result can be observed: when the natural growth rate *r* is low, the reward mechanism drives participants towards full cooperation. However, even with significant rewards for cooperation, the system’s resources will eventually be completely depleted, indicating that the resources are not sustainable. In this case, cooperative behaviour may lose its value owing to resource exhaustion. Therefore, it is advisable to avoid exploiting this type of common resource pool.

#### (ii) Moderately growing resource pool

**Figure 2.**
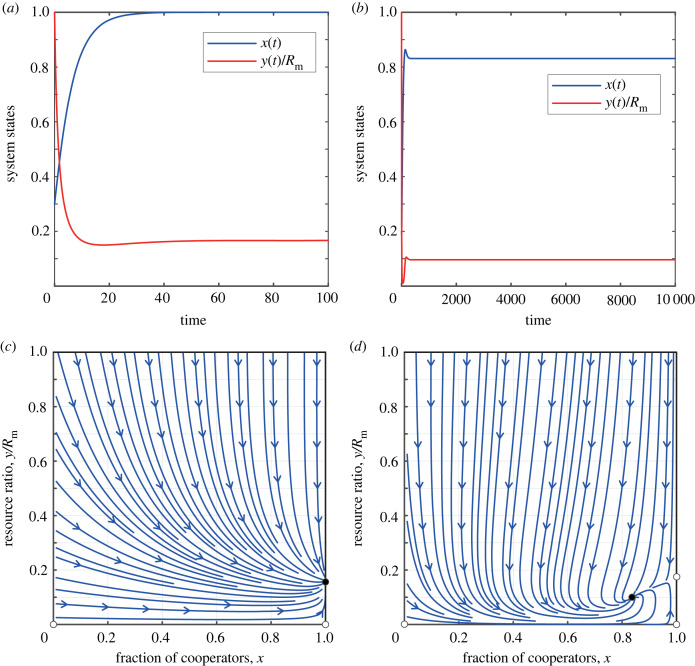
The coevolutionary dynamics of feedback-evolving game when the growth rate of the resource pool is moderate. (*a*,*c*) The evolution of the system’s state over time. (*b*,*d*) The phase diagram of *x* − *y*/*R*_m_. The parameters for (*a*) and (*c*) are: *r* = 0.6, *δ* = 0.2, *N* = 1000, *R*_m_ = 1000, *α* = 0.5 and *b*_m_ = 0.5. The parameters for (*b*) and (*d*) are: *r* = 0.6, *δ* = 0.02, *N* = 1000, *R*_m_ = 1000, *α* = 0.5 and *b*_m_ = 0.5.

In the case of moderate resource growth, when 0 < *E*_C_ < *r* < *E*_D_. We have $N\delta -\alpha b_{m}+(\alpha Nb_{m}^{2}/rR_{m})+(\alpha^{2}Nb_{m}^{2}/rR_{m})>0$. We find that, depending on the value of tax revenue *δ*, the system may stabilize at different fixed points. When $\delta -\alpha b_{m}+(\alpha Nb_{m}^{2}/rR_{m})>0$, the system has only three boundary fixed points, (0, 0), (1, 0), and (1, *R*_m_ − (*Nb*_m_/*r*)). By analysing the signs of the eigenvalues, we determine that, in this case, the system has a unique stable fixed point at (1, *R*_m_ − (*Nb*_m_/*r*)). It can be observed that when tax revenue *δ* is high, the system eventually leads to all participants transforming into cooperators who share the common resource pool, allowing sustainable development of the resource pool. This intriguing scenario unfolds vividly in the first column of figure 2.

In the event that the expression $\delta -\alpha b_{m}+(\alpha Nb_{m}^{2}/rR_{m})$ is less than zero, the system unfolds to reveal three boundary fixed points of notable significance: (0, 0), (1, 0) and (1, *R*_m_ − (*Nb*_m_/*r*)), as well as one interior fixed point (x*, y*). According to the sign analysis of eigenvalues, the first three boundary fixed points are unstable, while the last interior fixed point is stable (the stability proof for interior points can be found in the electronic supplementary material). It can be observed that when the tax revenue *δ* is low, the system will eventually reach a state of coexistence between cooperators and defectors. In this case, the resource environment can also be sustainably used. This situation is shown in the second column of figure 2.

#### (iii) Rapidly growing resource pool

In the case of rapid resource growth, when *r* > *E*_D_. We find that the choice of tax revenue *δ* is crucial. Depending on the different levels of tax revenue, the system may stabilize at different fixed points. However, owing to the high natural growth rate of resources, the resource level in the system will always be maintained at a relatively high level for sustainable development. The stability results of the system corresponding to different levels of tax revenue are provided below.

**Figure 3.**
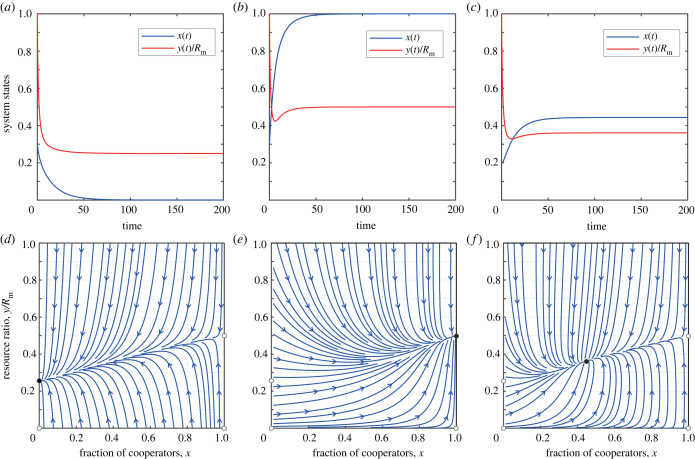
The coevolutionary dynamics of dynamic feedback games when the growth rate of the resource pool is high. (*a*–*c*) The evolution of the system’s state over time. (*d*–*f*) The phase diagram of *x* − *y*/*R*_m_. The parameters for (*a*) and (*d*) are *r* = 1.0, *δ* = 0.00002, *N* = 1000, *R*_m_ = 1000, *α* = 0.5 and *b*_m_ = 0.5. The parameters for (*b*) and (*e*) are *r* = 1.0, *δ* = 0.2, *N* = 1000, *R*_m_ = 1000, *α* = 0.5 and *b*_m_ = 0.5. The parameters for (*c*) and (*f*) are *r* = 1.0, *δ* = 0.04, *N* = 1000, *R*_m_ = 1000, *α* = 0.5 and *b*_m_ = 0.5.

When $N\delta -\alpha b_{m}+(\alpha Nb_{m}^{2}/rR_{m})+(\alpha^{2}Nb_{m}^{2}/rR_{m})<0$, the system has four boundary fixed points. These points are as follows: (*x*, *y*) = (0, 0), (1, 0), (1, *R*_m_ − (*Nb*_m_/*r*)) and (0, *R*_m_ − (*Nb*_m_/*r*)(1 + *α*)). Through our theoretical analysis, we ascertain that the fixed point (0, *R*_m_ − (*Nb*_m_/*r*)(1 + *α*)) is stable, while the other fixed points are unstable. This is an interesting result, as with low tax revenue *δ*, all participants in the system eventually become defectors. However, owing to the high natural growth rate of resources, the resource level in the system is maintained at a relatively high level for sustainable development. This situation is shown in the first column of figure 3.

When $\delta -\alpha b_{m}+(\alpha Nb_{m}^{2}/rR_{m})>0$, the system presents four boundary fixed points. These points are distinctly located at: (*x*, *y*) = (0, 0), (1, 0), (1, *R*_m_ − (*Nb*_m_/*r*)), (0, *R*_m_ − (*Nb*_m_/*r*)(1 + *α*)). It can be proven that the fixed point (1, *R*_m_ − (*Nb*_m_/*r*)) is stable, while the other fixed points are unstable. In this case, we can observe that when the tax revenue *δ* is relatively high, all participants in the system will eventually become cooperators, and the resource level in the system will be maintained at a relatively high level for sustainable development. This situation is shown in the second column of figure 3.

When $\delta -\alpha b_{m}+(\alpha Nb_{m}^{2}/rR_{m})<0<N\delta -\alpha b_{m}+(\alpha Nb_{m}^{2}/rR_{m})+(\alpha^{2}Nb_{m}^{2}/rR_{m})$, the system has an interior fixed point (x*, y*). Consequently, the system has four boundary fixed points: (*x*, *y*) = (0, 0), (1, 0), (1, *R*_m_ − (*Nb*_m_/*r*)), (0, *R*_m_ − (*Nb*_m_/*r*)(1 + *α*)) and one interior fixed point (x*, y*). Through analysis, we are able to ascertain that the interior fixed point (x*, y*) is stable (for the stability of interior fixed points, please refer to the electronic supplementary material), while the four boundary fixed points are unstable. In this case, we can observe that the system will eventually reach a state of coexistence between cooperators and defectors. In this state, the resource level in the system will be maintained at a relatively high level for sustainable development. This situation is shown in the third column of figure 3.

### (b) Tax-punishment

Drawing parallels to the scenario involving rewards, an examination of the tax-punishment strategy through the equation (1.7) unveils a similar pattern. The system has four boundary fixed points: (*x*, *y*) = (0, 0), (1, 0), (1, *R*_m_ − (*Nb*_m_/*r*)), (0, *R*_m_ − (*Nb*_m_/*r*)(1 + *α*)). The detailed existence conditions and stability analysis of the interior equilibrium points are presented in the electronic supplementary material. Based on the different natural growth rate *r*, we will discuss the following three cases.

#### (i) Slowly growing resource pool

**Figure 4.**
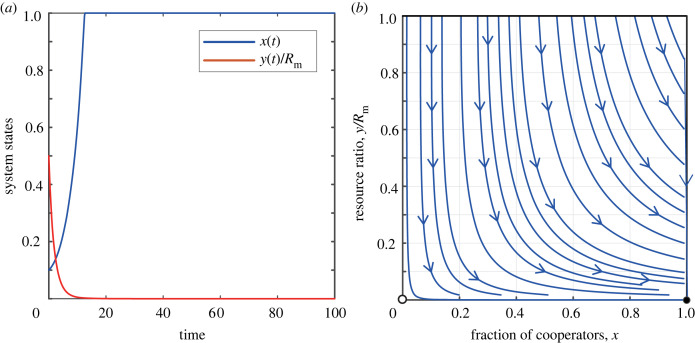
The coevolutionary dynamics of coupled systems, under scenarios of a slowly growing resource pool, incorporate tax-punishment. (*a*) The evolution of the system’s states over time. (*b*) The phase diagram of *x* − *y*/*R*_m_. Parameters are *r* = 0.25, *δ* = 0.2, *N* = 1000, *R*_m_ = 1000, *α* = 0.5 and *b*_m_ = 0.5.

In the case of slow resource growth, that is 0 < *r* < *E*_C_ < *E*_D_, the system has two equilibrium points: (0, 0) and (1, 0). Obviously, by analysing the signs of the eigenvalues, we can determine that (0, 0) is unstable, while (1, 0) is stable. Figure 4 unfurls the tapestry of time, charting the evolutionary trajectory of the proportion of cooperators and the abundance of the common resource pool under the condition where 0 < *r* < *E*_C_ < *E*_D_. This figure also presents the corresponding phase diagram. Similar to the case of tax-reward, when the natural growth rate *r* is low, the punishing mechanism drives the participants towards complete cooperation. However, even with significant penalties imposed on defectors, the resources will eventually be completely depleted. In this scenario, high levels of cooperation become meaningless.

#### (ii) Moderately growing resource pool

**Figure 5.**
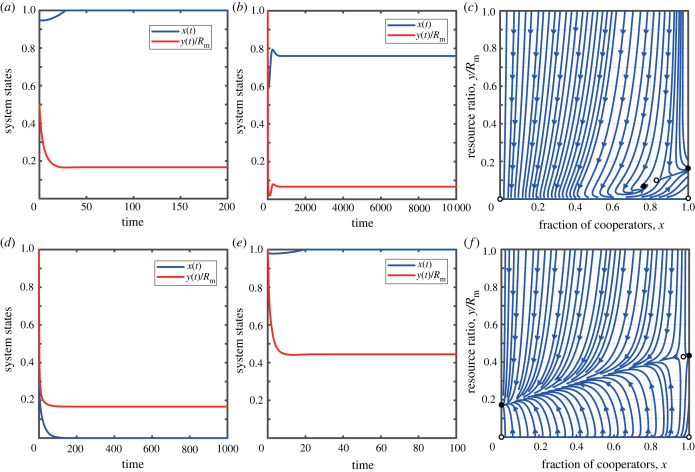
The coupled system can produce bistable outcomes. (*a*,*b*,*d*,*e*) The evolution of the system’s state over time. (*c*,*f*) The phase diagram of *x* − *y*/*R*_m_. The parameters for (*a*–*c*) are *r* = 0.6, *δ* = 0.004, *N* = 1000, *R*_m_ = 1000, *α* = 0.5 and *b*_m_ = 0.5. The parameters for (*d*–*f*) are *r* = 0.9, *δ* = 0.003, *N* = 1000, *R*_m_ = 1000, *α* = 0.5 and *b*_m_ = 0.5.

When resource growth is moderate, specifically 0 < *E*_C_ < *r* < *E*_D_. For the boundary fixed point, it is evident that (1, *R*_m_ − (*Nb*_m_/*r*)) is stable when $-N\delta +\alpha b_{m}-(\alpha Nb_{m}^{2}/rR_{m})<0$ (more detailed theoretical analysis can be found in the electronic supplementary material). Now we provide a numerical example. We find that the system has three boundary fixed points (0, 0), (1, 0), (1, *R*_m_ − (*Nb*_m_/*r*)) and two interior fixed points $\left(x_{1}^{*},y_{1}^{*}\right)$, $\left(x_{2}^{*},y_{2}^{*}\right)$. Let us assume $x_{1}^{*}<x_{2}^{*}$. In this situation, the system has two stable fixed points (1, *R*_m_ − (*Nb*_m_/*r*)) and $\left(x_{1}^{*},y_{1}^{*}\right)$, whereas the remaining fixed points are all unstable. This scenario is illustrated in the first row of figure 5. We find that there are five equilibrium points in the phase plane, among which there are two stable equilibria. One is located inside the phase plane, indicating that cooperation can be maintained at a relatively high level and the resource level can also remain constant. The other is located on the right boundary of the phase plane, implying that all individuals in the group choose cooperative behaviour while resources can be sustained. It should be noted that the emergence of one of the evolutionary outcomes depends on the initial levels of cooperation and resources.

#### (iii) Rapidly growing resource pool

When the natural growth rate is high, specifically *r* > *E*_D_. Clearly, when $-N\delta +\alpha b_{m}-(\alpha Nb_{m}^{2}/rR_{m})<0$, the fixed point (1, *R*_m_ − (*Nb*_m_/*r*)) is stable; when $\delta -\alpha b_{m}+(\alpha Nb_{m}^{2}/rR_{m})+(\alpha^{2}Nb_{m}^{2}/rR_{m})<0$, the fixed point (0, *R*_m_ − (*Nb*_m_(1 + *α*)/*r*)) is stable (detailed theoretical analysis is presented in the electronic supplementary material). Here, we provide a numerical example that ensures the existence of an unstable interior equilibrium point in the coupled system. As depicted in the second row of figure 5, there exist five equilibrium points in the phase plane: (0, 0), (1, 0), (1, *R*_m_ − (*Nb*_m_/*r*)), (0, *R*_m_ − (*Nb*_m_(1 + *α*)/*r*)), and one interior fixed point (x*, y*). In this situation, the two boundary fixed points (1, *R*_m_ − (*Nb*_m_/*r*)) and (0, *R*_m_ − (*Nb*_m_(1 + *α*)/*r*)) are stable, but the interior fixed point (x*, y*) is unstable. Similar to the scenario with a moderate natural growth rate, the system displays bistable outcomes, underscoring the importance of the selection of initial conditions, as the system’s stability hinges on these chosen values. Despite the potential for the system to ultimately evolve towards either complete cooperation or complete defection, the high natural growth rate of the common resource pool enables the system to consistently maintain a non-zero level of resources.

It is worth noting that the coupled system can generate limit cycle dynamics. Electronic supplemetary material, figure S1 shows the representative evolution of cooperation level and abundance of the common resource pool over time, as well as the phase diagram of *x* − *y*/*R*_m_ under the condition of *E*_C_ < *r* < *E*_D_. We observe four equilibrium points in the phase plane, of which three are boundary equilibrium points and one is an interior equilibrium point, all of which are unstable. Trajectories originating from any initial point in the phase plane ultimately converge to a limit cycle within the plane (figure S1B). This implies that the frequency of cooperators and the level of resources exhibit oscillating evolutionary dynamics. The trajectory of system states over time in figure S1A also verifies this result.

## 4. Monte Carlo simulations

In order to corroborate the efficacy of our model, we embarked on a series of Monte Carlo numerical simulations. The application of Monte Carlo methodologies within the framework of system dynamics serves as a powerful tool to augment our comprehension of system behaviour, and more importantly, to bolster our predictive prowess. This, in turn, equips decision-makers with the capacity to devise more potent strategies and to appraise the risks and uncertainties intertwined with their decisions with greater precision. In the forthcoming section, we will elucidate the robustness and effectiveness of our dual-model framework, as substantiated by Monte Carlo simulations.

### (a) Tax-reward

Each individual *i* in the population is required to pay tax *δ*, which will be evenly distributed as reward to cooperators. Each individual has only two choices: to become a cooperator (denoted as *s*_*i*_ = 1) or become a defector (denoted as *s*_*i*_ = 0). If individual *i* is a cooperator, his/her payoff is $F_{i}=b_{m}y(t)/R_{m}-\delta +N\delta /\sum_{i=1}^{N}s_{i}$. Otherwise, if individual *i* is a defector, his/her payoff is *F*_*i*_ = (1 + *α*)*b*_m_*y*(*t*)/*R*_m_ − *δ*. The update of the common resource level *y*(*t*) is governed by the following rule:

4.1 $$y(t+1)=y(t)+ry(t)\left(1-\frac{y(t)}{R_{m}}\right)-\sum_{i=1}^{N}\left(s_{i}\frac{y(t)b_{m}}{R_{m}}+(1-s_{i})\frac{y(t)b_{m}(1+\alpha )}{R_{m}}\right).$$

The selection of individual strategies involves each individual *i* learning from a random individual *j*. If the payoff *F*_*i*_ for individual *i* under his/her chosen strategy is lower than the payoff *F*_*j*_ for individual *j*, then individual *i* will adopt the strategy of *j* with a probability *q*. Otherwise, individual *i* will remain with his/her current strategy. We refer to the probability *q* as the transition probability:

4.2 $$q=\frac{F_{\;j}-F_{i}}{M},$$

where *M* quantifies the uncertainty in strategy adoption, and when *q* > 1, we set *q* = 1.

**Figure 6.**
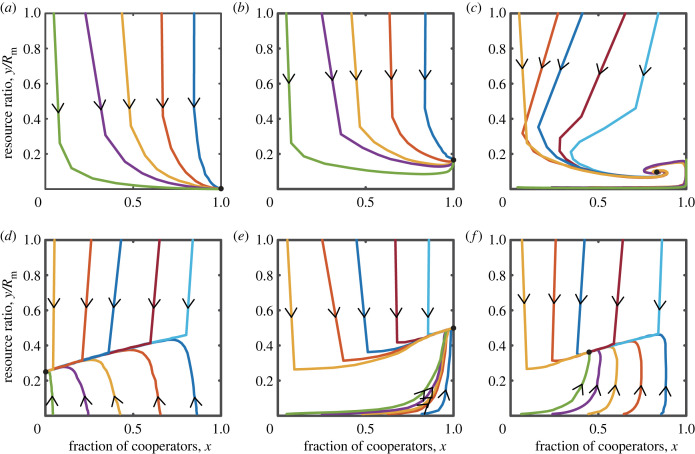
The results of Monte Carlo simulations for the coupled system with tax-reward under three distinct scenarios. The solid black dots represent the calculated stable fixed points. (*a*) The simulation outcomes when the resource growth rate is low. (*b*,*c*) Two different sets of results for scenarios with a moderate resource growth rate. (*d*–*f*) Three distinctly varied simulation outcomes under conditions of a high resource growth rate. The parameters for (*a*) are *M* = 1, *r* = 0.25, *δ* = 0.2, *N* = 1000, *R*_m_ = 1000, *α* = 0.5 and *b*_m_ = 0.5. The parameters for (*b*) are *M* = 1, *r* = 0.6, *δ* = 0.2, *N* = 1000, *R*_m_ = 1000, *α* = 0.5 and *b*_m_ = 0.5. The parameters for (*c*) are *M* = 0.1, *r* = 0.6, *δ* = 0.02, *N* = 1000, *R*_m_ = 1000, *α* = 0.5 and *b*_m_ = 0.5. The parameters for (*d*) are *M* = 1, *r* = 1, *δ* = 0.00002, *N* = 1000, *R*_m_ = 1000, *α* = 0.5 and *b*_m_ = 0.5. The parameters for (*e*) are *M* = 1, *r* = 1, *δ* = 0.2, *N* = 1000, *R*_m_ = 1000, *α* = 0.5 and *b*_m_ = 0.5. The parameters for (*f*) are *M* = 1, *r* = 1, *δ* = 0.04, *N* = 1000, *R*_m_ = 1000, *α* = 0.5 and *b*_m_ = 0.5.

Figure 6 illustrates the coevolutionary dynamics under different scenarios: slow growth, moderate growth and rapid growth, as obtained from Monte Carlo simulations. From the figure, it can be observed that the system eventually stabilizes at the theoretically calculated fixed points. In the case of slow resource growth, the magnitude of rewards is insufficient to sustain resource development. Although all participants eventually transition to cooperators, the resources will be completely depleted (figure 6*a*). Under moderate resource growth, results consistent with the theoretical expectations are obtained, where the stability of the system is determined by the magnitude of rewards (figure 6*b*,*c*). In the scenario of rapid resource growth, depending on the reward levels, the system may reach a state of complete defection (figure 6*d*), complete cooperation (figure 6*e*), or coexistence of cooperation and defection (figure 6*f*). Irrespective of which of the aforementioned three scenarios manifests, the resources in the public pool consistently maintain a stable level.

### (b) Tax-punishment

For the tax-punishment strategy, if individual *i* is a cooperator, his/her payoff is *F*_*i*_ = *b*_m_*y*(*t*)/*R*_m_ − *δ*. Otherwise, if individual *i* is a defector, his/her payoff is $F_{i}=(1+\alpha )b_{m}y(t)/R_{m}-\delta -N\delta /\sum_{i=1}^{N}(1-s_{i})$. We still adopt the same updating strategy for the common resource pool and individual strategies as in the case of reward.

**Figure 7.**
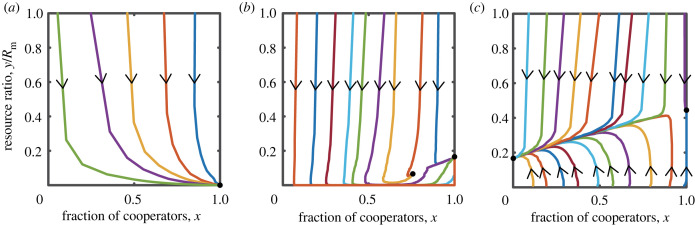
Monte Carlo simulation results of the feedback-evolving game model under the tax-punishment scenario when considering (*a*) low, (*b*) moderate and (*c*) high resource growth rates. The solid black dots in the figures represent the calculated stable fixed points. The parameters for (*a*) are *M* = 1, *r* = 0.25, *δ* = 0.2, *N* = 1000, *R*_m_ = 1000, *α* = 0.5 and *b*_m_ = 0.5. The parameters for (*b*) are *M* = 2, *r* = 0.6, *δ* = 0.004, *N* = 1000, *R*_m_ = 1000, *α* = 0.5 and *b*_m_ = 0.5. The parameters for (*c*) are *M* = 1, *r* = 0.9, *δ* = 0.003, *N* = 1000, *R*_m_ = 1000, *α* = 0.5 and *b*_m_ = 0.5.

In figure 7, we present the agent-based simulation results considering scenarios with low, moderate and high growth rates of the public resource pool, respectively, when taking into account tax-punishment. Our simulation results corroborate the theoretical findings: when the growth rate of the public pool is low, despite all individuals in the group ultimately opting for a cooperative strategy, the resources inevitably reach a state of complete depletion (figure 7*a*). This implies that even universal cooperation cannot overcome the tragedy of the commons induced by an excessively low resource growth rate. When the growth rate of the public pool is moderate, depending on the initial conditions, the system either stabilizes in a state where cooperators and defectors coexist sustainably with resources, or stabilizes in a state where all individuals choose cooperation accompanied by sustainable resources (figure 7*b*). Either outcome results in a situation where both cooperation and resources are maintained. When the growth rate of the public pool is high, another bistable outcome can emerge: regardless of whether the population ultimately evolves into a state of complete cooperation or complete defection, resources can be sustained (figure 7*c*). Owing to the high natural growth rate of resources in this case, the resource level of the system can be sustained at a higher level and be developed sustainably.

## 5. Conclusion

The pivotal importance of sustainable resource development is gaining heightened acknowledgement in our contemporary society. Consequently, forecasting the enduring impacts of individual actions on resource stewardship has come to the forefront as a paramount challenge. Existing research has explored the complex relationship between resource environments and stakeholder behaviours, leading to the formulation of sophisticated models [47–53]. These models integrate diverse factors such as incentives [31], regulatory norms [54] and sociocultural influences [55], providing a comprehensive perspective on resource utilization dynamics. Although relevant research has been initiated, the understanding of how to better design incentives to regulate individual behaviours for promoting resource sustainability remains in a nascent stage.

We have established tax-reward and tax-punishment models to more accurately describe the reality of resource development. We have found that the effects generated by rewarding cooperators and punishing defectors are not uniform. Specifically, in scenarios characterized by a low natural growth rate of resources, neither the tax-reward nor the tax-punishment model is capable of achieving sustainable resource development solely through rewarding cooperators or punishing defectors. Regardless of the magnitude of rewards given to cooperators or punishments imposed on defectors, although participants in the system tend to become all cooperators, the resources in the environment will eventually deplete. Therefore, for resources with low natural growth rates, more effective protection measures should be implemented, such as avoiding exploitation, to prevent eventual resource depletion. For resources with high natural growth rates, our results in the tax-reward model have demonstrated that sustainable resource development can be ensured. Here, the magnitude of rewards does not affect the sustainability of resources but only the total resource level in the steady state. This provides important insights for practical applications: by adjusting the size of the tax payment *δ*, the system can eventually stabilize at a higher resource level. Regarding the tax-punishment model, our findings suggest the potential for bistability within the system. The stability of the fixed points is not solely tethered to the magnitude of the tax payment, denoted as *δ*, but also intricately intertwined with the choice of the initial state. This dual dependency adds a layer of complexity to the system’s dynamics. In this case, choosing an appropriate initial state is also critical in maintaining a high resource level. For a resource pool with moderate natural growth rates, our results in the tax-reward model show that the system only exhibits one stable fixed point. In reality, we need to adjust the tax revenue *δ* to maintain a higher resource level in the system. In our exploration of the tax-punishment model, we have discovered that the system may display bistability, suggesting that the system’s stability is contingent upon the strength of punishment and the selection of the initial states. However, it is important to acknowledge that in the tax-punishment model, scenarios may arise where stable fixed points are absent. For instance, this paper presents an example of a limit cycle, wherein the system fails to achieve a stable state and instead undergoes periodic cycling. This phenomenon could stem from intricate internal interactions and feedback loops within the system. This insight is crucial for gaining a more profound understanding of the system’s dynamic behaviour and for predicting future evolutionary trends.

Our results provide important theoretical foundations for decision-making in practical contexts, aiding us in better resource management and utilization to achieve sustainable development. However, we also recognize that the model has its inherent limitations. For instance, we assume that all individuals are perfectly rational, while, in reality, individual decision-making may be influenced by various factors. Therefore, future research can further expand and optimize our models, such as introducing more real-life factors, considering irrationality in individual behaviour [56–58], accounting for resource growth rates varying with the current resource level *y*, or incorporating the migration of individuals [59,60]. It would also be beneficial to compare the actual cost-effectiveness of the tax-reward and tax-punishment mechanisms proposed in our current work. Optimal institutional incentives for promoting cooperation, as studied in recent works within the context of public goods and Prisoner’s Dilemma games, have shown that adaptive rewarding strategies considering population statistics, such as the threshold number of cooperators in the population, or local network properties, like the number of cooperators in the network, could be more cost-efficient in promoting cooperation [61–65]. Exploring the applicability of such adaptive approaches in our current context, depending on the resource growth rate and resource dynamics, could provide further insights into effective incentive mechanisms for sustainable resource management.

## Data Availability

Source code is available at Dryad: https://doi.org/10.5061/dryad.pnvx0k6w6 [66]. Supplementary material is available online [67].
